# The acute physiological status of white sharks (*Carcharodon carcharias*) exhibits minimal variation after capture on SMART drumlines

**DOI:** 10.1093/conphys/coz042

**Published:** 2019-08-13

**Authors:** R D Tate, B R Cullis, S D A Smith, B P Kelaher, C P Brand, C R Gallen, J W Mandelman, P A Butcher

**Affiliations:** 1 National Marine Science Centre, Southern Cross University, PO Box 4321, Coffs Harbour, New South Wales, Australia; 2 National Institute of Applied Statistics Research Australia, Faculty of Engineering and Information Sciences, University of Wollongong, Wollongong, New South Wales, Australia; 3 NSW Department of Primary Industries, National Marine Science Centre PO Box 4321, Coffs Harbour, New South Wales, Australia; 4 Anderson Cabot Center for Ocean Life, New England Aquarium, 1 Central Wharf, Boston, MA, USA

**Keywords:** Aspartate aminotransferase, bather protection, capture stress, elasmobranch, lactate, shark bite management

## Abstract

Drumlines incorporating SMART (Shark-Management-Alert-in-Real-Time) technology are a new tool used in several bather protection programmes globally. In New South Wales (NSW), Australia, the white shark (*Carcharodon carcharias*) is a target species for SMART drumlines because they are often involved in attacks on humans. To understand white shark sensitivity to capture and to establish protocols around acceptable timeframes for responding to alerts, 47 juvenile and subadult white sharks were caught on SMART drumlines at five locations off the east coast of Australia. There was no at-vessel mortality during the sampling period. After capture, blood was sampled from each shark to assess its acute physiological status. Of the 18 metabolites investigated, only lactate and aspartate aminotransferase exhibited significant positive relationships with the capture duration on SMART drumlines. These results indicate that the capture process is relatively benign and that the current response times used here are appropriate to minimize long-term negative impacts on released white sharks. Where white sharks are likely to interact negatively with beachgoers, SMART drumlines can therefore be a useful addition to bather protection programmes that also aim to minimize harm to captured animals. Other shark species captured on SMART drumlines should also be investigated to gain broader understanding of potential physiological consequences of using this new technology.

## Teaser

White sharks (Carcharodon carcharias) are implicated in many bites on people. Subsequently, they have become a target species for shark bite management strategies. SMART drumlines allow fast responses to, and releases of, captured animals. This research shows that white sharks’ immediate physiological response to capture on SMART drumlines is minimal.

## Introduction

Over the last two decades, reports of unprovoked shark bites on humans have increased by approximately 80% globally (Shark Research Institute, 2019). While infrequent, with approximately six human fatalities annually worldwide, these events are traumatic and are often highly publicized (McCagh *et al*., 2015). Management responses often result in the implementation of bather protection strategies (Curtis *et al*., 2012; Fraser-Baxter and Medvecky, 2018), with measures such as mesh nets, drumlines, aerial- and land-based surveillance, physical barriers and a number of sensory deterrents (Dudley, 1997; Oelofse and Kamp, 2006; Reid *et al*., 2011; Sumpton *et al*., 2011; Hart and Collin, 2015; Colefax *et al*., 2018; Gray and Gray, 2017). However, traditional fishing methods like mesh nets and drumlines are often lethal for targeted and bycatch species (Dudley *et al*., 1998; Cliff and Dudley, 2011; Brazier *et al*., 2012; Gibbs and Warren, 2015). They can also impact threatened, endangered and protected species (Curtis *et al*., 2012; Pepin-Neff and Wynter, 2018a, b). The lethality of these methods has led to the development of, and preference for, less invasive strategies including bather education and nonlethal technologies (Pepin-Neff and Wynter, 2018a, b; Simmons and Mehmet, 2018).

Traditional drumlines have been used as part of bather protection programmes globally (Dudley *et al*., 1998; Sumpton *et al*., 2011; Hazin and Afonso, 2014). One of the main factors leading to death during capture using drumlines or similar fishing gear is the length of time an animal is hooked (Butcher *et al*., 2015). A range of sublethal effects on a captured animals physiological status can lead to hyperglycaemia, lactic acidosis and hyperkalemia, which may indicate post-release mortality of animals found alive on drumlines or as bycatch in commercial fisheries (Mandelman and Skomal, 2009; Skomal and Mandelman, 2012). In 2013, a modified version of the traditional drumline using the “Catch-A-Live”® system was developed, making the lines SMART (Shark-Management-Alert-in-Real-Time as named in NSW) (Guyomard *et al*., 2019). These SMART drumlines use a GPS-enabled buoy in conjunction with traditional drumlines to provide near real-time alerts to the presence of a captured animal, allowing for fast response times that may reduce the likelihood of injurious consequences for captured animals.

While physiological status associated with various forms of capture is still being characterized in elasmobranchs, interspecific variation is apparent and may inform species-specific conservation efforts (Skomal and Mandelman, 2012). There is a range of known secondary physiological stress responses in elasmobranchs that may indicate deleterious impacts of capture (Skomal and Mandelman, 2012). For example, elevated blood potassium known as hyperkalemia (Mandelman and Farrington, 2007; Frick *et al*., 2012; Butcher *et al*., 2015; Dapp *et al*., 2017), elevated blood glucose known as hyperglycaemia (Frick *et al*., 2010; Skomal and Mandelman, 2012; Bouyoucos *et al*., 2018), and elevated blood lactate (Wells and Davie, 1985; Gallagher *et al*., 2014; French *et al*., 2015) commonly accompany mortality after elasmobranchs are exposed to various types of capture. These, and a suite of other blood variables, were analysed in this study due to their prominence in past research and known relationships with capture. This range of variables was also evaluated to provide a broad assessment of physiological status in this first study examining the response of white sharks to capture. Managing stress and minimizing mortality are particularly important for the conservation of elasmobranchs due to their relatively late sexual maturation, long gestation and typically small litters compared with other marine fishes (Hoenig and Gruber, 1990; Dulvy *et al*., 2014; Pardo *et al*., 2016).

White sharks (*Carcharodon carcharias*), tiger sharks (*Galeocerdo cuvier*) and bull sharks (*Carcharhinus leucas*) are the three primary species considered potentially harmful to humans. Subsequently, these species are targeted in an Australian trial utilizing SMART drumlines. This programme aims to mitigate shark–human interactions, minimize bycatch and to quantify the post-release movements of tagged sharks. While tiger and bull sharks are commercially fished in NSW, white sharks are listed as vulnerable in Australia under NSW and Australian law (*NSW Fisheries Management Act* 1994 and the *Environment Protection and Biodiversity Conservation Act 1999*, respectively), and under international conventions of the United Nations (i.e. The Convention on International Trade in Endangered Species, EPBC 1999; and the Convention on the Conservation of Migratory Species of Wild Animals, CITES 2017). White sharks are thought to be responsible for more than a third of the shark bites in NSW since 2000. Despite this, target sharks and all other species caught on SMART drumlines are released alive and in good condition. However, little is known about the impact of capture on their physiological status. Because white sharks are both protected and a target of bather protection strategies, it is vital that we understand how capture may impact their physiological status.

To explore the association between the stress response of captured animals and the amount of time spent hooked, this study aimed to quantify the acute physiological status of juvenile and subadult white sharks after being caught using SMART drumlines and secured to the research vessel prior to release. To do this, we analysed a range of whole-blood metabolites, including some that have not previously been measured in this species. This information will help to develop a conservative range of response times to ensure relatively low physiological impact for animals captured by SMART drumlines and later released.

## Material and methods

Blood samples were collected over a 13-month period between July 2016 and August 2017 at five locations on the north and mid-north coast of NSW, Australia: Lennox Head/Ballina (−28.8108 S 153.6103 E); Evans Head (−29.1111 S 153.4388 E); Coffs Harbour (−30.3103 S 153.1556 E); Crowdy Head (−31.8368 S 152.7478 E); and Forster (−32.1748 S 152.5181 E). Sampling was opportunistic, capitalizing on the capture of white sharks on SMART drumlines as part of a broader tagging and tracking research programme (*Unpublished data*, P. Butcher NSW DPI). White sharks were sampled on 52 occasions for 47 individuals (five recaptured sharks were sampled).

### Fishing gear and animal capture

The same SMART drumline configurations were used throughout the trial. This included an anchor (either comprising 3 m of 10 mm Ø galvanized chain by itself or with a 4.5-kg Danforth sand anchor on the end), and 20 m of 10 mm Ø polypropylene (PP) rope and an A1 polyform anchor buoy (279 × 381 mm). A second (surface) line (2.0 m of 10 mm Ø elasticised cord) was attached to a SMART buoy (model number MLI-s) and then a holding line of 0.5 m of 10 mm Ø PP rope and a larger A3 polyform drumline buoy (432 × 584 mm). A shock sleeve (incorporating two 1.1 m lengths of elasticised cord (10 mm Ø) encased in herring-bone material) and trace, which was either a 1.6 or a 3.2 m wire cable (3.0 mm Ø either plain or covered in poly-vinyl chloride, PVC) suspended from the buoy with a 20/0 circle hook (Mustad©) at the end. Each hook was constructed of 9 mm Ø duratin-coated carbon steel wire with shaft, bend and gape lengths of 122, 113 and 56 mm, respectively. Lines were all were baited with ~ 0.75–1 kg Sea Mullet (*Mugil cephalus*). A 2.0-m monofilament ‘trigger line’ (2.0 mm Ø) was attached between the elasticized cord and the SMART buoy. When the hook was bitten, the trigger line separated the magnet from the socket in the SMART buoy, and a signal was transmitted via satellite, alerting researchers via SMS, telephone call, and email.

On each fishing day, SMART drumlines were deployed during daylight hours at ~ 500 m from shore and waters ~ 6–15 m deep. Once an alert was received, a vessel travelled to the SMART drumline to monitor the gear and the sharks' activity. While no formal process was undertaken, each shark was approached once it maintained a normal upright swimming position without heavily thrashing. Once the SMART drumline was retrieved, the trace was attached to a longer rope so the shark could be secured to the side of the vessel using an additional (i) cross-pectoral fin rope and (ii) tail rope which contained a PVC sleeve to minimize abrasions.

### Sample extraction and laboratory analysis

Following Butcher *et al*., (2015), white sharks were sampled for whole blood (5–8 ml) once they were brought alongside the boat. Blood was extracted by caudal venepuncture using a 90 mm, 18-gauge needle and a 10-ml syringe. A small amount (~10 μm) of whole blood was immediately tested in an Accutrend Plus field sampler (Roche Diagnostics, Australia) for lactate and glucose (Wells and Pankhurst, 1999; Awruch *et al*., 2011; Butcher *et al*., 2015). The remaining blood was transferred to an 8-ml plasma separator tube containing lithium heparin (BD Vacutainer), stored on ice temporarily, and centrifuged at 5000 rpm for 4 min. Plasma samples were separated into three 2-ml vials and frozen immediately at −18°C in the field before being transferred to a −80°C freezer in a laboratory. Samples were analysed by IDEXX Laboratories (Brisbane, Australia) within 7 days to prevent blood chemistry alterations (Barton, 2002; Butcher *et al*., 2015), using a Beckman Coulter AU680 automated system. A total of 18 variables were assessed from the blood of each shark (Table 1).

**Table 1:** Descriptive results and Wald *p* values in the baseline model for duration of time on the line, sex and fork length of the blood-chemistry variables for 47 (and five recaptures) white sharks (*Carcharodon carcharias*) sampled after capture from SMART drumlines between May 2016 and August 2017

Blood variable	Mean ± SE	Range	Time on the line	Sex	Fork length
Albumin (mmol l^−1^)	3.2 ± 0.1	1.0–5.0	0.032	0.463	0.315
Alkaline phosphatase (IU l^−1^)	15.5 ± 1.0	3.0–37.0	0.238	0.542	**<0.001**
Anion gap (mmol l^−1^)	7.7 ± 0.9	−15.0—30.2	0.205	0.222	0.329
Aspartate aminotransferase (IU l^−1^)	19.0 ± 1.4	1.0–58.0	**0.006**	0.085	0.085
Bicarbonate (mmol l^−1^)	5.1 ± 0.3	1.0–9.0	0.018	0.167	0.067
Calcium – unionized (mmol l^−1^)	3.6 ± 0.1	2.8–5.8	0.073	0.296	0.482
Chloride (mmol l^−1^)	252.9 ± 3.1	198.0–335.0	0.394	0.356	0.429
Cholesterol (mmol l^−1^)	1.3 ± 0.1	0.4–2.3	0.547	0.726	0.079
Creatine kinase (IU l^−1^)	81.6 ± 20.4	2.0–1003	0.098	**0.005**	0.896
Globulin (mmol l^−1^)	19.3 ± 0.4	12.0–24.0	0.641	0.325	0.109
Glucose (mmol l^−1^)	5.4 ± 0.1	3.9–6.8	0.179	0.567	0.552
Lactate (mmol l^−1^)	12.3 ± 0.6	4.8–26.9	**<0.001**	0.874	0.259
Magnesium (mmol l^−1^)	2.1 ± 0.4	0.9–14.5	0.027	0.600	0.723
Potassium (mmol l^−1^)	4.2 ± 0.1	2.4–7.2	0.188	0.595	0.136
Sodium (mmol l^−1^)	261.1 ± 2.5	205.0–322.0	0.199	0.205	0.565
Total inorganic phosphate (mmol l^−1^)	2.4 ± 0.1	1.7–3.1	0.738	0.731	0.065
Total protein (g l^−1^)	22.5 ± 0.4	13.0–29.0	0.457	0.376	0.202
Urea (mmol l^−1^)	370.2 ± 3.8	281.4–432.4	0.377	0.298	0.655

Significant values are highlighted using bold, noting the conservative significant level of *p* < .01.

### Data collected

Environmental data associated with the capture of each shark were also collected and where applicable included in the predictive models. These included the average and maximum wind speeds (km h^−1^), wind direction (as a bearing), barometric pressure (hPa), sea state (Beaufort scale 1–5), water temperature (°C), cloud cover (scored as 0–8, with 0 being no cloud and 8 complete cloud cover), turbidity/visibility (scored as 0 muddy to 5 clear), average and maximum swell and sea height (m), humidity (%), time of sunrise and sunset, time of moon rise and set and the moon phase (% visible). Following blood sampling, the sex and length (fork—FL to the nearest cm) of each shark were measured, along with time of capture (h:m), duration of time spent on the hook (minutes), and capture location (town and beach).

### Statistical modelling

To determine the terms to be included in the linear mixed model (LMM), the approach of Smith and Cullis (2017) was used, with the sampling design factors (plot factors) as ‘date,’ ‘location,’ ‘beach’ and ‘time of capture,’ with 28, 5, 11 and 5 levels, respectively. These factors, when considered together, uniquely index the observational units, which are the blood samples taken for each of the 52 captures (47 individual sharks plus five recaptured). There was a total of 36 unique drumlines (indexed by the combinations of the first three plot factors) with 10 of these having more than one capture (to a maximum of five). There was sufficient information to consider a crossed classified plot structure given by the following:

$$\begin{align*} &\mathrm{Date}\ast \left(\mathrm{Location}/\mathrm{Beach}/\mathrm{Time}\ \mathrm{of}\ \mathrm{capture}\right)=\mathrm{Date}\\ &\quad+\mathrm{Location}+\mathrm{Location}:\mathrm{Beach}+\mathrm{Date}:\mathrm{Location}\\ &\quad+\mathrm{Date}:\mathrm{Location}:\mathrm{Beach}+\mathrm{Date}:\mathrm{Location}:\mathrm{Beach}\\ &\,\quad:\mathrm{time}\ \mathrm{of}\ \mathrm{capture}. \end{align*}$$

The final term indexed the observational units to form the treatment structure given by the following:

$$\begin{align*} &1+\mathrm{time}\ \mathrm{on}\ \mathrm{hook}+\mathrm{FL}+\mathrm{water}\ \mathrm{temperature}+\mathrm{water}\ \mathrm{depth}\\ &\quad+\mathrm{sex}+\mathrm{time}\ \mathrm{of}\ \mathrm{capture}. \end{align*}$$

These treatment and plot structures formed the working generalized LMM (GLMM) where all model terms in the plot structure are assumed to be ‘random’ terms, while terms in the treatment structure were fitted as ‘fixed’ terms as follows:

$$\begin{align*} \mathrm{Fixed}&=\sim 1+\mathrm{time}\ \mathrm{on}\ \mathrm{hook}+\mathrm{FL}+\mathrm{water}\ \mathrm{temperature}\\ &\quad+\mathrm{water}\ \mathrm{depth}+\mathrm{sex}+\mathrm{time}\ \mathrm{of}\ \mathrm{capture}. \end{align*}$$

$$\begin{align*} \mathrm{Random}&=\sim \mathrm{date}+\mathrm{location}+\mathrm{location}:\mathrm{beach}\\ &\quad+\mathrm{date}:\mathrm{location}+\mathrm{date}:\mathrm{location}:\mathrm{beach}\\ &\quad+\mathrm{date}:\mathrm{location}:\mathrm{beach}:\mathrm{time}\ \mathrm{of}\ \mathrm{capture}. \end{align*}$$

The working GLMM was then extended to incorporate recaptures. Five animals were recaptured once, and this was accounted for by including an additional random term ‘tag,’ with 47 levels.

To examine the data for the presence of interactions between variables in the model, the base-case LMM was extended to include the additional five first-order interaction terms in the ‘fixed’ model formula. A conservative approach for the significance of these terms was assessed using the approach of Benjamini and Hochberg (1995) to control the overall false discovery rate at a level of *p* < .01. A similar approach was used for assessing the significance of ‘time on the line after hooking,’ ‘sex’ and ‘fork length’ for all the traits.

All analyses were conducted in the R package ASReml-R which fits LMMs using the residual maximum likelihood. Inference for fixed effects was conducted using approximate Wald-type pivots (Butler *et al*., 2017) using a Kenward–Roger adjustment for the appropriate denominator degrees of freedom for each Wald pivot. Where required, blood variables were log transformed for normalization.

## Results

### Catch results

SMART drumlines were deployed across a variety of depths {mean [±SD] = 8.86 ± 1.7 m}, ranging from 6.2 to 14.6 m, and water temperatures {mean [±SD] = 19.7 ± 0.9°C}, ranging from 18.3 to 22.2°C. On arrival at a hooked shark, researchers spent 1–22 min {mean [±SD] = 6.3 ± 4.7 min} evaluating each SMART drumline and shark before they could safely move in to restrain the individual next to the boat. Fifty-two white sharks (47 individuals, five recaptured) comprising 33 females {mean [±SD] = 229.3 ± 45.3 cm FL, ranging 139–335 cm FL} and 19 males {mean [±SD] = 227.7 ± 45.3 cm FL, ranging 160–268 cm FL} were sampled for blood 10–75 min {mean [±SD] = 29.8 ± 15.2 min} post-capture, inclusive of the handling time taken to secure the sharks before blood could be safely extracted. The elapsed time from when each shark was secured at the boat to when blood was collected ranged from 1 to 13 min {mean [±SD] 2.8 ± 1.9}.

**Figure 1:**
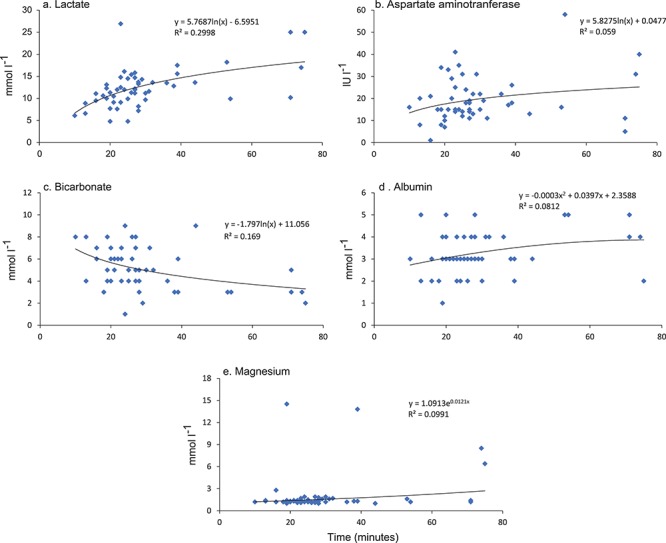
Significant relationships in GLMMs between the elapsed time after hooking and the concentrations of blood-chemistry variables in white sharks (*Carcharodon carcharias*) caught on SMART drumlines between May 2016 and August 2017 (*n* = 52)

### Blood variables

**Figure 2:**
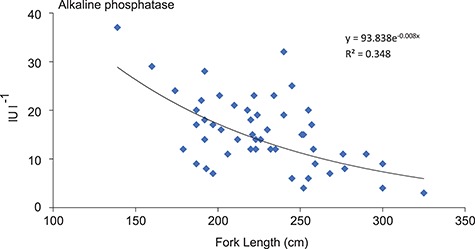
Significant relationships in GLMMs for fork length and alkaline phosphatase in white sharks (*Carcharodon carcharias*) caught on SMART drumlines between May 2016 and August 2017 (*n* = 52)

Descriptive results (mean ± SE and ranges) and the outcomes of the GLMMs of the blood variables collected from white sharks are shown in Table 1. No first-order interactions were significant, so we reverted to the base-case model. For the 18 blood variates analysed, only lactate (GLMM, *p* < .001, Table 1, Fig. 1a) and aspartate aminotransferase (GLMM, *p* < .01, Table 1, Fig. 1b) had significant positive relationships with the time spent on the line. Furthermore, non-significant trends were observed for bicarbonate (GLMM, *p* = .018), albumin (GLMM, *p* = .032) and magnesium (GLMM, *p* = .027) in relation to time spent on the line after hooking, noting our conservative significant level of .01 (Fig. 1c, d and e, respectively). For the biological variables, a similar approach for sex and fork length indicated a significant negative impact of fork length on alkaline phosphatase (GLMM, *p* < .001, Table 1, Fig. 2). Sex was only influenced by creatine kinase with males (142.8 ± 50.7 IU l^−1^) having significantly higher concentration than females (48.7 ± 11.89 lU l^−1^, *p* < .01, Table 1).

## Discussion

This is the first study to examine the physiological status of white sharks caught on SMART drumlines. The amount of time spent on the hook influenced the acute physiological status of white sharks, with concentrations of lactate and aspartate aminotransferase increasing significantly as capture time lengthened. However, the lack of significant effects for most of the blood variates indicates that the SMART drumline capture process does not exacerbate many of the measured variables. Furthermore, no at-vessel mortality was observed for capture durations up to 75 min, and all sharks were later detected as part of a broader tracking study (*Unpublished data*, P. Butcher NSW DPI). Therefore, the current requirement for contracted fishers and research staff to respond to an alert within 30 min may help mitigate deleterious physiological consequences for white sharks. SMART drumlines are an effective inclusion in shark bite mitigation strategies aiming for low impact on targeted animals, such as white sharks, and are a valuable research tool to study other large fishes.

The duration of a capture event often influences the level of physiological impact on elasmobranchs (Kneebone *et al*., 2013; Gallagher *et al*., 2014; Butcher *et al*., 2015). Similarly, the amount of time spent on a SMART drumline in the present study was the most influential covariate on white shark blood variables, with both lactate and aspartate aminotransferase (AST) increasing with capture time. Lactate commonly increases with time spent on a line (Marshall *et al*., 2012; Butcher *et al*., 2015; Dapp *et al*., 2016), indicating elevated anaerobic metabolism associated with fighting against the gear during capture (Renshaw *et al*., 2012). Although there is no direct scientific basis that elevated concentrations of blood lactate cause death in elasmobranchs, it has been shown to vary consistently across a range of different ‘fishing’ methods and species. Therefore, blood lactate concentration may provide a useful predictive indicator of animal mortality. For example, in the blue shark (*Prionace glauca*) caught on long lines, blood lactate concentrations were almost five times higher in moribund animals (~27.7 mmol l^−1^) than in those that were alive (Moyes *et al*., 2006). Bronze whaler sharks (*Carcharhinus brachyurus*) with a blood lactate concentration exceeding 27.4 mmol l^−1^ were predicted to die post-release after long-line capture (Dapp *et al*., 2016). Similarly, both sicklefin lemon sharks (*Negaprion acutidens*) and blacktip reef sharks (*Carcharhinus melanopterus*), respectively, experienced elevated blood lactate concentrations 6- and 14-times (to ~ 22 and 21 mmol l^−1^) baseline values after gill-net capture and air exposure (Bouyoucos *et al*., 2018).

The SMART drumline capture process also resulted in elevated levels of AST which is an enzyme typically found within internal organs and muscles. Its presence in the blood can indicate internal injury (Manire *et al*., 2001; Kori-Siakpere *et al*., 2010). A number of studies have reported elevated blood AST concentrations in various species after exposure to capture stressors, such as bull sharks caught in gill-nets (Manire *et al*., 2001), catfish exposed to pollutants (Oluah, 1999; Kori-Siakpere *et al*., 2010) and greynurse sharks (also known as sand tiger sharks, *Carcharias taurus*) that died in large mesh-nets (Otway, 2015). In elasmobranchs, amino acids play a role in gluconeogenesis and ketogenesis in the liver (Speers-Roesch and Treberg, 2010). AST catalyses a chemical reaction that results in ketone-bodies, which are a primary source of energy for elasmobranchs. In the present study, the elevated levels may be due to an increased energy demand associated with capture on SMART drumlines. Elasmobranch heart and skeletal muscles are unable to oxidize fatty acids, so this is done in the liver where lipids are stored; thus, they rely on ketone bodies for aerobic metabolism (Metcalf and Gemmell, 2005; Otway, 2015). This also explains why albumin is seldom found in the blood of elasmobranchs (Zammit and Newsholme, 1979; Ballantyne, 2016) and may be related to the elevated AST observed here as physiological demand forces access to energy stores and potentially results in intracellular damage. However, in a study examining the physiological stress responses of Atlantic stingrays (*Hypanus sabinus*) exposed to air, the authors concluded that this species may not use ketone bodies (β-hydroxybutyrate) for energy during acute stress (Lambert *et al*., 2018).

Without baseline physiological profiles, we cannot unambiguously assert that these two differences are a reflection of an altered physiological status directly attributable to acute capture-stress. However, previous studies have shown variable physiological states across species. For example, blue sharks caught with long lines and released alive had mean blood AST and lactate concentrations of 38.3 and 5.8 mmol l^−1^, respectively, compared with 26.9 and 27.7 mmol l^−1^ in moribund animals (Moyes *et al*., 2006). In that study, the amount of time spent hooked was more than 10 hr and capture depths were up to 100 m. This may explain the higher and highly variable AST concentrations when compared with the present study, as AST has been shown to be positively associated with capture depth in other shark species (Butcher *et al*., 2015). By comparison, blood AST concentrations in juvenile and subadult white sharks were more stable at ~ 19.0 mmol l^−1^, indicating that irreversible damage to these animals is unlikely and that the energy demand resulting from SMART drumline capture is comparatively low.

Blood lactate in white sharks in the present study was ~ 12.3 mmol l^−1^ (4.8–26.9 mmol l^−1^). Blacktip reef sharks had elevated blood lactate 3 hr after a 3-min gill-net capture and 1-min air exposure (Bouyoucos *et al*., 2018). This may suggest that the ‘burst swimming’ associated with capture evokes an immediate physiological response that continues through a period of recovery before placating (Brooks *et al*., 2012). Furthermore, species- and gear-specific variations in physiological responses to capture are likely related to different behaviour whilst hooked. Recent studies quantifying the behaviour of three shark species caught on drumlines used accelerometers to measure the intensity of the captured animals' behaviour over the length of a capture (Gallagher *et al*., 2017). Across all three species—blacktip sharks, (*Carcharhinus limbatus*), nurse sharks (*Ginglymostoma cirratum*), and tiger sharks—there was a significant positive relationship between plasma lactate concentration and maximum acceleration value, the majority of which occurred in the first 5 min of hooking. However, no relationship was detected between lactate and the length of time spent hooked. Similarly, Guida *et al*., (2016) found that gummy sharks (*Mustelus antarcticus*) experienced no significant variation in lactate as a result of time spent on simulated longlines, asserting that this is likely due to their ability to respire while stationary (also see Guida *et al*., 2017). Some of the white sharks in the present study were observed to swim away with strong bursts immediately after capture and occasionally thrashing and rolling before swimming steadily against the gear. Differences in behaviour such as the intensity with which a species resists capture, and the ability to respire without forward movement, may contribute to the overall physiological status of captured sharks and provide some species with a greater resilience to capture.

Although white sharks visually appeared to be in a relatively good state of health at the time they were sampled after capture on SMART drumlines, the lack of baseline physiological profiles for white sharks obviates firmer conclusions. One study that quantified the mortality of juvenile and subadult white sharks after net capture reported high survival rates (92.9%) of sharks found alive, for which the mean soak-time was 29.5 hr, compared with 40.7 hr for dead sharks (Lyons *et al*., 2013). These high survival rates support our assertion that white sharks are robust to the relatively short SMART drumline capture process.

### Conclusion

We observed no significant change in the majority of physiological variables as a result of time spent on the line for juvenile and subadult white sharks caught on SMART drumlines. The relatively benign effect of SMART drumline capture on the white sharks indicates that this species can readily tolerate a response time of at least 30 min. However, post-release fitness implications cannot be predicated solely on these results. Future research should include all targeted sharks and a range of common bycatch species (especially protected species) to expand our knowledge about the species-specific physiological impacts of SMART drumline capture. It should be noted that, relative to other published studies, the maximum amount of time spent on the line was regulated by the standard procedure of the NSW Shark Management Strategy. To test the impacts of capture more broadly, it would be beneficial to test the physiological status after longer capture times. This may offer insights into similar stressors faced by fishes as a result of other bite-management programs as well as commercial and recreational fisheries.
