# Corrigendum to: The acute physiological status of white sharks (*Carcharodon carcharias*) exhibits minimal variation after capture on SMART drumlines

**DOI:** 10.1093/conphys/coz090

**Published:** 2019-12-10

**Authors:** R D Tate, B R Cullis, S D A Smith, B P Kelaher, C P Brand, C R Gallen, J W Mandelman, P A Butcher

**Affiliations:** 1 National Marine Science Centre, Southern Cross University, PO Box 4321, Coffs Harbour, New South Wales 2450, Australia; 2 National Institute of Applied Statistics Research Australia, Faculty of Engineering and Information Sciences, University of Wollongong, Wollongong, New South Wales 2522, Australia; 3 NSW Department of Primary Industries, National Marine Science Centre PO Box 4321, Coffs Harbour, New South Wales 2450, Australia; 4 Anderson Cabot Center for Ocean Life, New England Aquarium, 1 Central Wharf, Boston, MA 02110, USA

This manuscript has been amended to correct details in Table 1, Calcium: Potassium ratio (IU l−1) was recorded as a variable name in the table, when this should have been Creatine Kinase (IU l^−1^). This has now been corrected. As a result of this a sentence in the Results section has also been amended to read “Sex influenced creatine kinase with males (142.8 ± 50.74 IU l^−1^) having significantly higher concentrations than females (48.7 ± 11.89 IU l^-1^, *p* < 0.01).” The authors apologize any confusion caused.

